# Supplementary material for: Actionable absolute risk prediction of atherosclerotic cardiovascular disease based on the UK Biobank
Source: PLoS One. 2022 Feb 11;17(2):e0263940. doi: 10.1371/journal.pone.0263940 (PMC8836294; doi:10.1371/journal.pone.0263940)
Supplement: S3 Table — (PDF) [file pone.0263940.s004.pdf]

S3 Table. Specifications of the python (v3.9.6) libraries and their versions used in this study.

Library Name	Version
cycler	0.10.0
future	0.18.2
imbalanced-learn	0.8.0
imblearn	0.0
joblib	0.16.0
kiwisolver	1.3.1
matplotlib	3.4.2
numpy	1.21.1
pandas	1.3.0
Pillow	8.3.1
pip	21.1.3
pyparsing	2.4.7
python-dateutil	2.8.1
python-dotenv	0.18.0
pytz	2020.1
Scikit-learn (1)	0.24.2
scipy	1.7.1
seaborn	0.11.1
setuptools	57.0.0
shap	0.40.0
six	1.15.0
threadpoolctl	2.1.0
xgboost (2,3)	1.4.2

1: Pedregosa F, Varoquaux G, Gramfort A, Michel V, Thirion B, Grisel O, et al. Scikit-learn: Machine Learning in Python. Journal of Machine Learning Research. 2011;12(85):2825–30.

2. Chen T, Guestrin C. XGBoost: A Scalable Tree Boosting System. Proceedings of the 22nd ACM SIGKDD International Conference on Knowledge Discovery and Data Mining. 2016 Aug 13;785–94.

3. XGBoost Documentation — xgboost 1.6.0-dev documentation [Internet]. [cited 2021 Nov 8]. Available from: https://xgboost.readthedocs.io/en/latest/
