# Supplementary material for: Actionable absolute risk prediction of atherosclerotic cardiovascular disease based on the UK Biobank
Source: PLoS One. 2022 Feb 11;17(2):e0263940. doi: 10.1371/journal.pone.0263940 (PMC8836294; doi:10.1371/journal.pone.0263940)
Supplement: S4 Table — (PDF) [file pone.0263940.s005.pdf]

S4 Table. List of utilized open-source methods, best parameters and references.

Method name and references	Best performing parameters*
Extreme Gradient Boosting (XGBoost) (1-3)	subsample: 0.8, n_estimators: 1500, max_depth: 5, learning_rae': 0.01, gamma: 1, colsample_bytree: 0.6
Logistic Regression with L1 regularization and saga solver (4,5)	solver=saga, max_iter=1000, penalty='l1'
Random Forest Classifier (4,6)	n_estimators=20, criterion='entropy', max_depth=None, min_samples_split=2, min_samples_leaf=1, max_features='auto'
The following classifiers were only briefly explored:	
Decision Tree Classifier (7)	criterion='entropy', splitter='best', max_depth=None, min_samples_split=2
Voting Classifier 1	voting='soft', combining the best Logistic Regression model with Random Forest Model with 1000 trees
Voting Classifier 2	voting='soft', combining the best Logistic Regression model with Random Forest Model with 20 trees
Multi-Layer Perceptron Classifier 1 (8)	activation='relu', solver='adam', alpha=0.0001, batch_size='auto', power_t=0.5, max_iter=1000,shuffle=True, tol=0.0001, verbose=False, warm_start=False, n_iter_no_change=10, early_stopping=True, validation_fraction=0.2, learning_rate_init=0.001, hidden_layer_sizes=(200,)
Multi-Layer Perceptron Classifier 2 (8)	activation='relu', solver='lbfgs', alpha=0.0001, power_t=0.5, tol=0.0001, verbose=False, warm_start=False, max_fun=15000, max_iter=1000, hidden_layer_sizes=(100,)
Multi-Layer Perceptron Classifier 3 (8)	activation='relu', solver='sgd', alpha=0.0001, batch_size='auto', learning_rate='adaptive', power_t=0.5, max_iter=1000, shuffle=True, tol=0.0001, verbose=False, warm_start=False,

	momentum=0.9, nesterovs_momentum=True, early_stopping=True, validation_fraction=0.2, learning_rate_init=0.1, hidden_layer_sizes=(200, 150)
Stochastic Gradient Descent Classifier (9,10)	loss='modified_huber', penalty='l2', max_iter=1000, learning_rate='optimal', n_jobs=-1
Ada Boost (11,12)	n_estimators=50, learning_rate=1.0
Gradient Boosting (13)	n_iter_no_change=None, tol=0.0001, learning_rate=0.1,, n_estimators=100, max_depth=3, subsample=1.0
K Neighbors Classifier (14)	algorithm='auto', p=2, n_jobs=-1, n_neighbors=5,
Quadratic Discriminant Analysis (15)	
Gaussian Naive Bayes (16,17)	

* Best performing parameters found using Grid Search, default parameters were used if not stated otherwise

1. Friedman JH. Greedy Function Approximation: A Gradient Boosting Machine. The Annals of Statistics. 2001;29(5):1189–232.

2. Chen T, Guestrin C. XGBoost: A Scalable Tree Boosting System. Proceedings of the 22nd ACM SIGKDD International Conference on Knowledge Discovery and Data Mining. 2016 Aug 13;785–94.

3. XGBoost Documentation — xgboost 1.6.0-dev documentation [Internet]. [cited 2021 Nov 8]. Available from: https://xgboost.readthedocs.io/en/latest/

4. Pedregosa F, Varoquaux G, Gramfort A, Michel V, Thirion B, Grisel O, et al. Scikit-learn: Machine Learning in Python. Journal of Machine Learning Research. 2011;12(85):2825–30.

5. McCullagh P, Nelder JA. Generalized linear models. Routledge; 2019.

6. Breiman L. Random Forests. Machine Learning. 2001 Oct 1;45(1):5–32.

7. Breiman L, Friedman JH, Olshen RA, Stone CJ. Classification And Regression Trees. Boca Raton: Routledge; 2017. 368 p.

8. Hinton GE. 20 - CONNECTIONIST LEARNING PROCEDURES11This chapter appeared in Volume 40 of Artificial Intelligence in 1989, reprinted with permission of North-Holland Publishing. It is a revised version of Technical Report CMU-CS-87-115, which has the same title and was prepared in June 1987 while the author was at Carnegie Mellon University. The research was supported by contract N00014-86-K-00167 from the Office of Naval Research and by grant IST-8520359 from the National Science Foundation. In: Kodratoff Y, Michalski RS, editors. Machine Learning [Internet]. San Francisco (CA): Morgan Kaufmann; 1990 [cited 2022 Jan 10]. p. 555–610. Available from: https://www.sciencedirect.com/science/article/pii/B9780080510552500298
9. Hearst MA, Dumais ST, Osuna E, Platt J, Scholkopf B. Support vector machines. IEEE Intell Syst Their Appl. 1998 Jul;13(4):18–28.
10. Zhang T. Solving large scale linear prediction problems using stochastic gradient descent algorithms. In: Proceedings of the twenty-first international conference on Machine learning [Internet]. New York, NY, USA: Association for Computing Machinery; 2004 [cited 2021 Nov 12]. p. 116. (ICML '04). Available from: https://doi.org/10.1145/1015330.1015332
11. Freund Y, Schapire RE. A Decision-Theoretic Generalization of On-Line Learning and an Application to Boosting. J Comput Syst Sci. 1997 Aug 1;55(1):119–39.
12. Hastie T, Rosset S, Zhu J, Zou H. Multi-class AdaBoost. Stat Interface. 2009;2(3):349–60.
13. Friedman JH. Greedy Function Approximation: A Gradient Boosting Machine. Ann Stat. 2001;29(5):1189–232.
14. Omohundro SM. Five balltree construction algorithms. International Computer Science Institute Berkeley; 1989.
15. Srivastava S, Gupta MR, Frigyik BA. Bayesian quadratic discriminant analysis. J Mach Learn Res. 2007;8(6).
16. Pedregosa F, Varoquaux G, Gramfort A, Michel V, Thirion B, Grisel O, et al. Scikit-learn: Machine Learning in Python. J Mach Learn Res. 2011;12(85):2825–30.
17. Zhang H. The optimality of naive Bayes. AA. 2004;1(2):3.
