# Supplementary material for: Actionable absolute risk prediction of atherosclerotic cardiovascular disease based on the UK Biobank
Source: PLoS One. 2022 Feb 11;17(2):e0263940. doi: 10.1371/journal.pone.0263940 (PMC8836294; doi:10.1371/journal.pone.0263940)
Supplement: Attachment — Submitted filename: Response to reviewers.pdf [file pone.0263940.s007.pdf]

Ajay Kesar
Ada Health GmbH
Karl-Liebknecht-Str. 1
10178 Berlin, Germany
science@ada.com

Dear Mr. Gadekallu, January 14, 2022

We thank the editor and the two reviewers for their comments on our manuscript. Below is our revised competing interests statement and responses to each point raised by the academic editor and reviewers. We hope that we satisfyingly addressed them and that the manuscript will be now suited for publication.

Academic editor:

1. Please ensure that your manuscript meets PLOS ONE's style requirements, including those for file naming.

We have modified the file naming to comply with the style requirements and are now fully compliant with the style requirements.

2. Competing Interests Statement:

We added the first and last sentence to our revised competing interests statement, for further clarification:

”This research was funded by Ada Health GmbH and has been conducted using the UK Biobank under application number 34802.”

And

“This does not alter our adherence to PLOS ONE policies on sharing data and materials.”

Full revised competing interests statement:

“This research was funded by Ada Health GmbH and has been conducted using the UK Biobank under application number 34802. All of the authors are or were employees of, contractors for, or hold equity in Ada Health GmbH. AK, AB, OB, HH, MJ, DN, BLS and SG are employees or company directors of Ada Health GmbH and some of the listed authors hold stock options in the company. Ada Health GmbH has received research grant funding from the Bill & Melinda Gates Foundation, Fondation Botnar, the Federal Ministry of Education and Research Germany, the Federal Ministry for Economic Affairs and Energy Germany and the European Union. PW is employed by Wicks Digital Health Ltd, which has received funding from Ada Health, AstraZeneca, Baillie Gifford, Biogen, Bold Health, Camoni, Compass Pathways, Coronna, EIT, Endava, Happify, HealthUnlocked, Inbeeo, Kheiron Medical, Lindus Health, Sano Genetics, Self Care Catalysts, The Learning Corp, The Wellcome Trust, THREAD Research, VeraSci, and Woebot. HH is the topic driver of the AI-based symptom assessment group of the WHO/ITU Focus Group on AI4H (Artificial Intelligence for Health) and SG is a member of the clinical evaluation topic group of the WHO/ITU Focus Group on AI4H.

A related patent application is currently pending with the title “System and method for predicting the risk of a patient to develop an atherosclerotic cardiovascular disease” and application number EP21191089.8.
This does not alter our adherence to PLOS ONE policies on sharing data and materials.”

3. Patent Mention in Competing Interests:

We have declared the requested name and number of the pending patent in the competing interests statement and added the last sentence for further clarification:

“This does not alter our adherence to PLOS ONE policies on sharing data and materials.”.

4. **Data Availability**

Please find below our revised data availability statement:

“There are restrictions prohibiting the provision of data in this manuscript. The data were obtained from a third party, UK Biobank, upon application. Interested parties can apply for data from UK Biobank directly, at http://www.ukbiobank.ac.uk. UK Biobank will consider data applications from bona fide researchers for health-related research that is in the public interest. By accessing data from UK Biobank, readers will be obtaining it in the same manner as we did.”

Reviewer #1:

1. The architecture looks very abstract and misses very important details. I recommend authors elaborate design and experimental setup of the proposed approach. The authors have described the materials and methods section, but I recommend including a detailed experimental setup for a better understanding and interpretation of the proposed work. A detailed, layered design describing the proposed approach should be included for a better understanding of readers.

Thank you for your feedback. We have included a new figure 1 on page 12 to describe the design and experimental setup of our approach.

The following sentence was modified for better visibility on page 12:

“Details on the used Python libraries, methods and parameters are provided in the supplementary data (S3 and S4 Tables)” and this sentence added: “Fig 1 visualizes an overview of all performed steps of our experimental setup.”

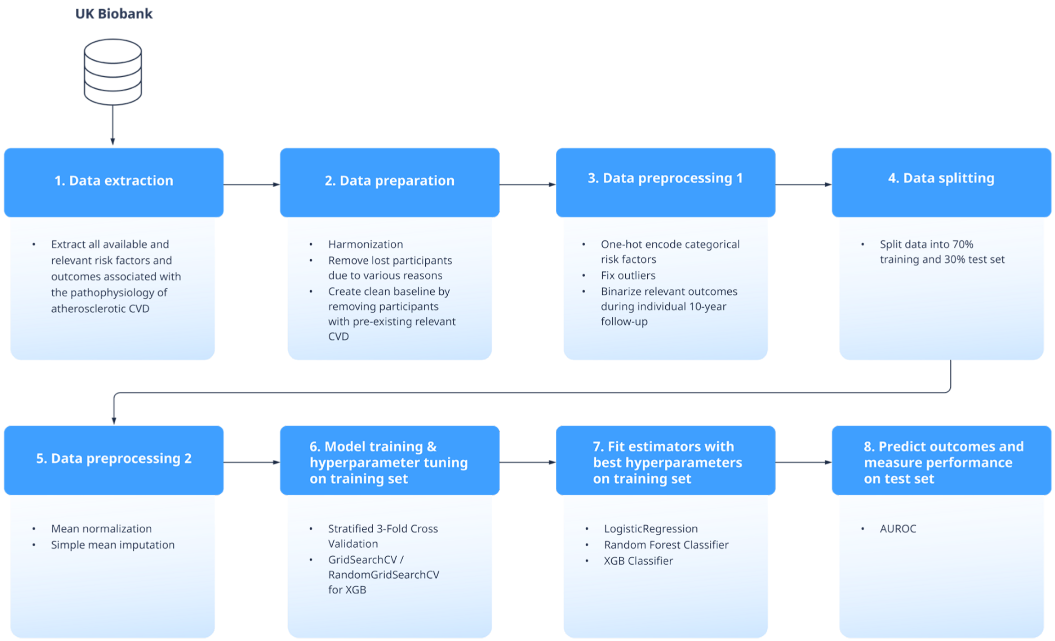

2. The authors have included 54 references (which occupies a lot of space), which has some unnecessary references which can be removed and essential references such as, https://www.frontiersin.org/articles/10.3389/fpubh.2021.762303/full", "https://ieeexplore.ieee.org/abstract/document/9170666/" can be referred.

We thank the reviewer for their suggestions of relevant literature. We can confirm that both studies are relevant as well and are now referenced in our manuscript. We referenced the first study twice and the second study once. As PLOS One is an online journal we understand there is not a strict limit on the number of references, but are happy to follow guidance from the editorial staff.

3. The results and discussions about how the proposed approach enhances the state of the art is missing. I recommend authors to highlight the contribution of the proposed work separately, along with the limitations of the system.

We have taken this suggestion into account and extended our discussion section on page 25 to highlight the contribution of our proposed model more clearly:

"Our atherosclerotic CVD prediction model has the potential to support healthcare systems by identifying more people at risk earlier and more accurately than currently available models and intervening with personalized behavior change programs. Currently available models, like Framingham and QRisk3, have limited predictive capability for atherosclerotic CVDs as they were not trained on all of them and do not provide actionable results."

4. The authors have not discussed the security and privacy aspects of the proposed system.

Thank you for highlighting this important missing aspect. We added the following remarks for completeness on page 26:

“A system and method gathering personal health data and predicting an individual's atherosclerotic CVD risk is handling sensitive health data (e.g. laboratory values) and must adhere to local regulations and best practices in data transfer, processing and storage to ensure data privacy and security.”

5. All tables should be symmetrical and should follow a similar formatting style.
All the equations should be written using a professional equation editor and should use a similar formatting style and numbering.
Check the entire manuscript for grammatical and typo errors.

Thank you for your feedback. We refined all table formatting styles to be more consistent. The whole manuscript was double checked by a native English speaker for grammatical and typographical errors.

Reviewer #2:

1. Abstract: please mention results of study in this section.

We thank the reviewer for their suggestion and have added the results to the abstract on page 1. While doing so, we also noticed a copy and paste error for the confidence intervals of our best performing Logistic Regression model which we have corrected.

2. Title: I think second part of the title can be reduced and integrated with first part

Thank you for your feedback. We shortened the title to “Actionable absolute risk prediction of atherosclerotic cardiovascular disease based on the UK Biobank” on the author page.

3. Introduction: why this study is new and novel. Please mention it in the introduction.

We appreciate the reviewer’s feedback on that matter and have modified and emphasized our unique contributions with a new second to last paragraph in the Introduction section on page 5:

“The aim of this study was to use a large-data ML approach to develop an actionable absolute risk prediction tool which takes into account the holistic health of an individual. Uniquely, we focussed on behavioral risk factors relating to all atherosclerotic CVD outcomes. Our goal was to have a holistic understanding of an individual's current health status, to better quantify their risk of atherosclerotic CVDs, and to provide actionable advice. Our approach is novel in that we employ a highly holistic understanding of an individual’s current health status, to better quantify their risk of all athersclerotic CVDs. By utilizing a comprehensive set of lifestyle factors, we enable the subsequent suggestion of personalized and actionable advice relating to unhealthy risk factors. Instead of using only a limited set of risk factors, we aimed to achieve this by taking multiple biological layers into account, which include: (i) multi-omics data from blood samples (e.g. lipidome and proteome); (ii) family history (e.g. genome), (iii) lifestyle data, (iv) clinical data and (v) environmental data; along with (vi) an extensive set of risk factors and outcomes.”

4. It is recommended to insert a workflow in the methodology section. Moreover, please describe method briefly in first paragraph of the method.

Thank you for your recommendation. We have included a new figure 1 on page 12 to describe the design and experimental setup of our approach in the methodology section.

The following sentence was added on page 12: “Fig 1 visualizes an overview of all performed steps of our experimental setup.”.

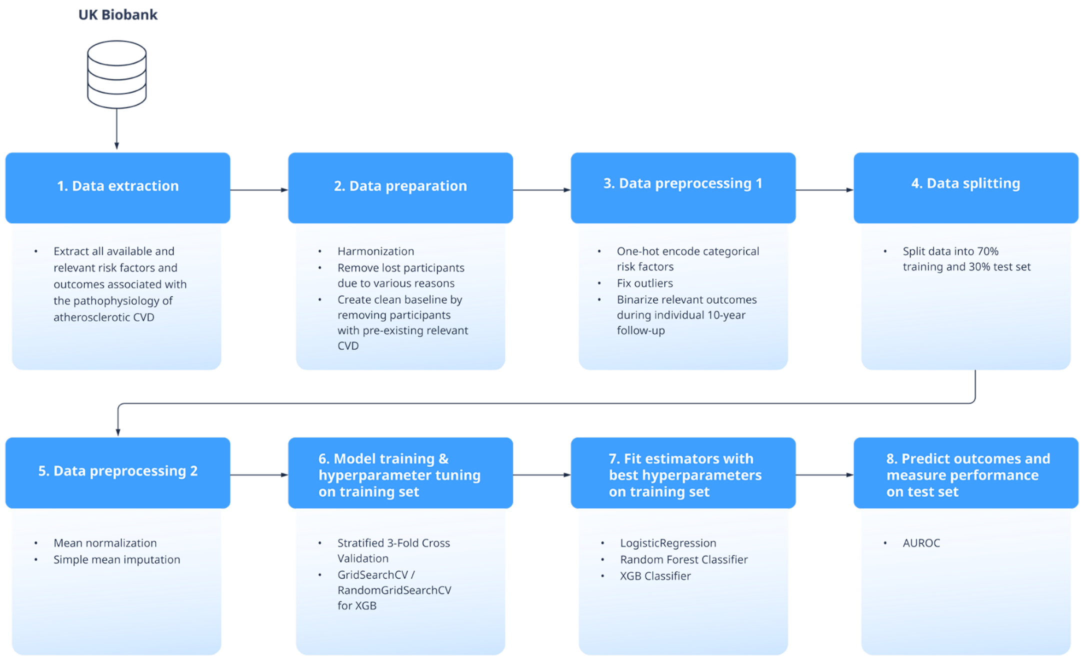

We also added a new brief summary to the methods section on page 6:

“Baseline data from the UK Biobank was utilized to extract an extensive set of risk factors and outcomes associated with the pathophysiology of atherosclerotic CVDs. A benchmarking pipeline was used to train and evaluate different standard and ML algorithms for the task of 10-year atherosclerotic CVD risk prediction. The performance was measured using AUROC and compared against the baseline models Framingham and QRisk3, which are widely used and recommended models. We evaluated our best performing models further by analysing the most informative features and assessed model generalizability and created a reduced model.”.

5. I cannot understand why these machine learning approaches were employed.

We certainly want to clarify for our readers why we have employed a ML approach and thank you for the opportunity to expand on our rationale in the text. Specifically, we added further clarifications to the method section on page 10:

“Since the introduction of the classic CVD risk prediction methods, the field of supervised machine learning has developed from classical statistics with the sole purpose of maximizing predictive accuracy with modern statistical methods. Therefore, in addition to using standard linear models, we tested the major ML approaches, covering a wide spectrum of the possible ML design space, to evaluate which model type performs best for our task. Based on our initial benchmarking pipeline results, we focused on reporting the results of the initially best performing models: logistic regression, random forest and XGBoost.”

6. I would like to know the selected parameters for running each machine learning approach. It is necessary to change parameters and achieve accuracy result. In fact, a sensitivity analysis should be performed.

Thanks for your feedback. We have added additional information to address your point in the supplementary file S4 Table "List of utilized open-source methods, best parameters and references", and here we have provided the parameters of the 3 benchmarked methods. For better visibility, we modified the following sentence on page 12:

"Details on the used Python libraries, methods and parameters are provided in the supplementary data (S3 and S4 Tables)."

We also added the parameters of the other tested methods to the data supplement file S4 Table.

Additionally, we performed a sensitivity analysis for our best performing Logistic Regression model using Shapley Additive Explanations (SHAP values) and provided the full analysis as a new figure in the supplementary data S1 Figure.

We added this sentence to the statistical paragraph of the methods section on page 13:

[...] "and performed a sensitivity analysis using Shapley Additive Explanations (SHAP values) for the best performing linear model"

and added the following sentences to the manuscript on page 20:

"We provided a sensitivity analysis using SHAP values of the best performing Logistic Regression model for all risk factors in the supplementary materials (S1 Fig.)"

and on the last page 35:

"**S1 Fig. Shapley Additive Explanations (SHAP value) of each risk factor for the logistic regression model.** (PNG) This summary plot combines risk factor importance with risk factor effects. It shows the relationship between the value of a risk factor and its impact on the prediction. Risk factors are sorted according to their importance along the y-axis. Each point in the summary plot is a Shapley value for a risk factor and an instance. The position of a Shapley value on the y-axis is determined by the risk factor importance and on the x-axis by the Shapley value. The color represents the value of a risk factor from low to high. Overlapping points are jittered on the y-axis direction, showing the distribution of the Shapley values per risk factor."

We hope these modifications satisfyingly increase the quality of our manuscript.

Sincerely on behalf of all authors,

Ajay Kesar
